# Supplementary material for: Ablation of glypican-3 enhances radiosensitivity in liver cancer by prolonging G2/M arrest and activating the ATM/CHK2 pathway
Source: bioRxiv. 2026 May 14:2026.05.11.724294. Preprint. [Version 1] doi: 10.64898/2026.05.11.724294 (PMC13193009; doi:10.64898/2026.05.11.724294)
Supplement: Supplement 1 [file media-1.pdf]

[Supplementary materials]

Ablation of glypican-3 enhances radiosensitivity in liver cancer by prolonging G2/M arrest and activating the ATM/CHK2 pathway

Authors:
Joon-Yong Chung[1,*], Hima Makala[1], Woonghee Lee[1], Olivia W. Lee[2], Simran Khurana[3], Jeong Won Kim[4], Julia Sheehan-Klenk[1], Divya Nambiar[1], Stanley Fayn[1,5], Ayla O. White[3], Eun Joo Chung[3], Nada Alani[6], Sabrina Ramelli[6], Stephen M. Hewitt[6], Travis H. Stracker[3], Deborah E. Citrin[3], Peter L. Choyke[1], Freddy E. Escorcia[1,3*]

Affiliations:
[1] Molecular Imaging Branch, Center for Cancer Research, National Cancer Institute, National Institutes of Health, Bethesda, MD 20892, USA.
[2] Integrative Tumor Epidemiology Branch, Division of Cancer Epidemiology and Genetics, National Cancer Institute, National Institutes of Health, Bethesda, MD 20892, USA.
[3] Radiation Oncology Branch, Center for Cancer Research, National Cancer Institute, National Institutes of Health, Bethesda, MD 20892, USA.
[4] Department of Pathology, Kangnam Sacred Heart Hospital, Hallym University College of Medicine, Seoul, 07441, Republic of Korea
[5] Oxford Institute for Radiation Oncology, Department of Oncology, University of Oxford, Oxford, UK.
[6] Laboratory of Pathology, Center for Cancer Research, National Cancer Institute, National Institutes of Health, Bethesda, MD 20892, USA.

Corresponding authors: Joon-Yong Chung, Email: chungjo@mail.nih.gov and Freddy E. Escorcia, Email: freddy.escorcia@gmail.com

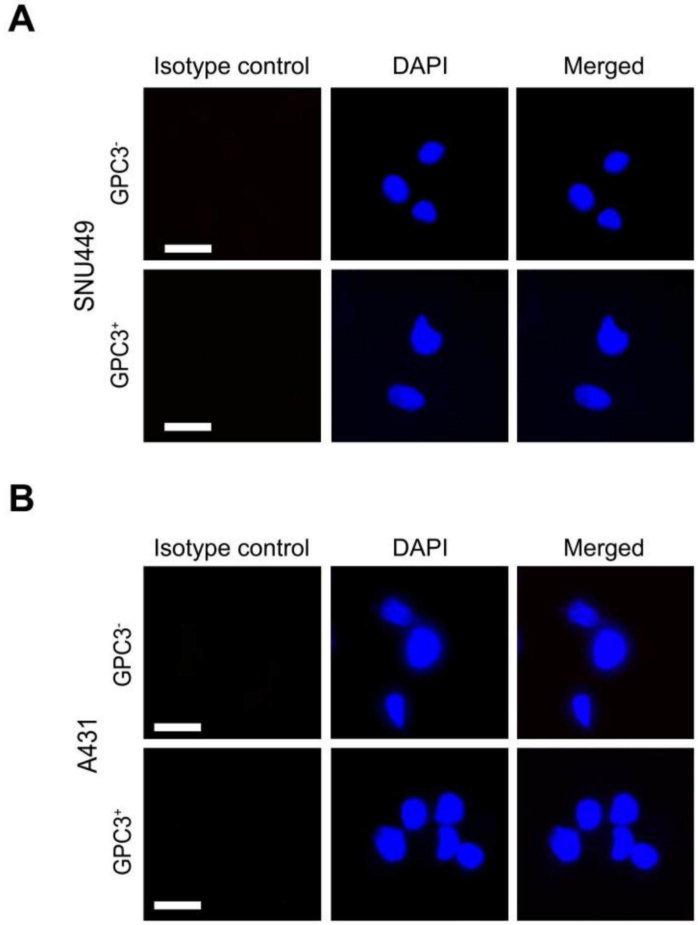

Supplementary Fig. S1. Immunofluorescence staining with isotype control antibody. The isotype control was negative in both SNU449/GPC3 (*A*) and A431/GPC3 (*B*) cells as well as in cells deficient in GPC3. Scale bars are shown for 50 μm.

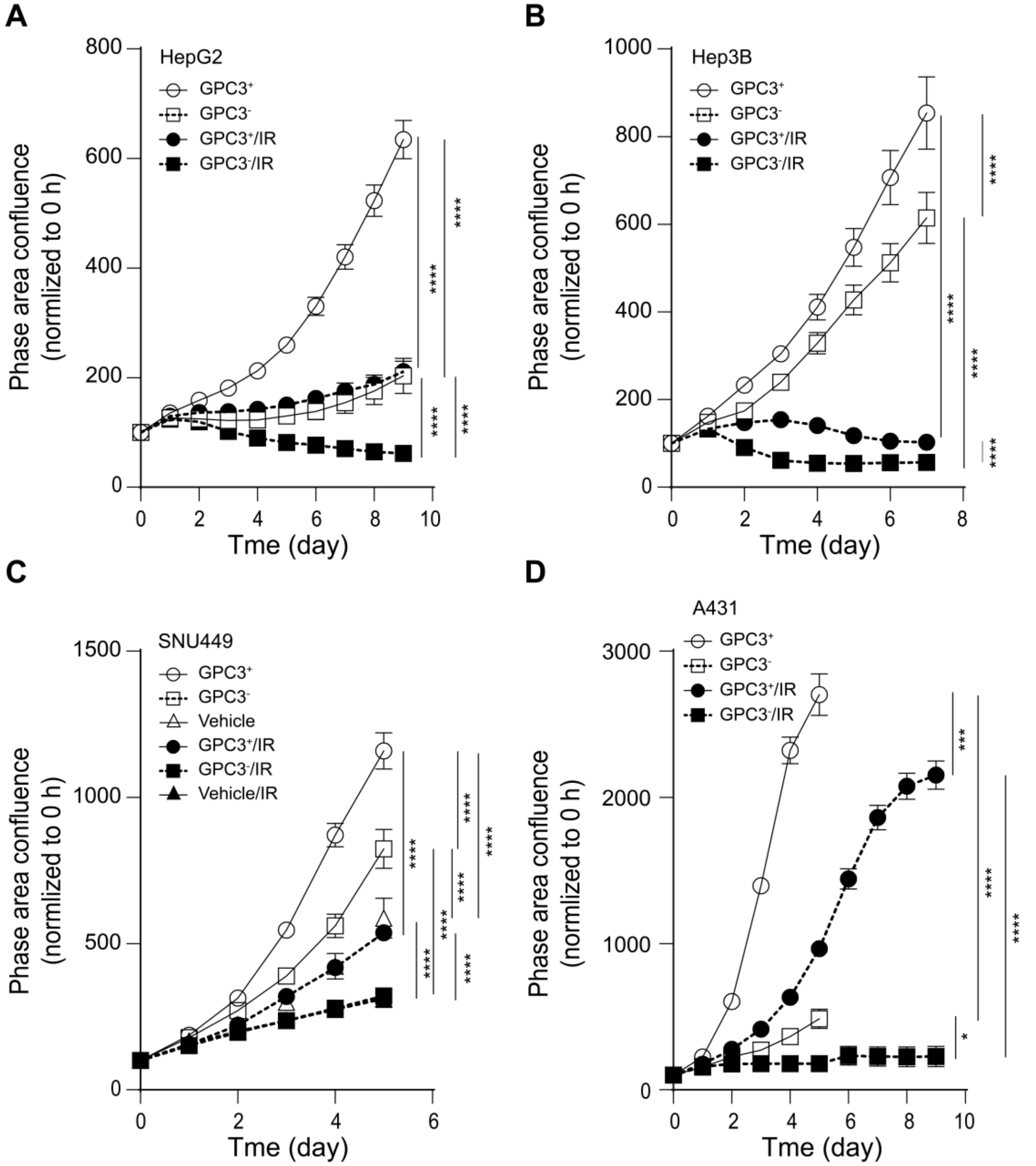

Supplementary Fig. S2. Proliferation analysis of GPC3$^+$ and GPC3$^-$ cancer cells under irradiated and non-irradiated conditions. Cell lines tested included HepG2 (*A*), Hep3B (*B*), SNU449 and vector control SNU449/V (*C*), and A431 (*D*), each with GPC3$^+$ and GPC3$^-$ variants. Cell growth was tracked using the IncuCyte® live-cell imaging system, both without treatment and after exposure to 6 Gy irradiation (IR). Statistical comparisons were made using two-way ANOVA followed by multiple comparisons. *p<0.05, ***p<0.001, ****p<0.0001.

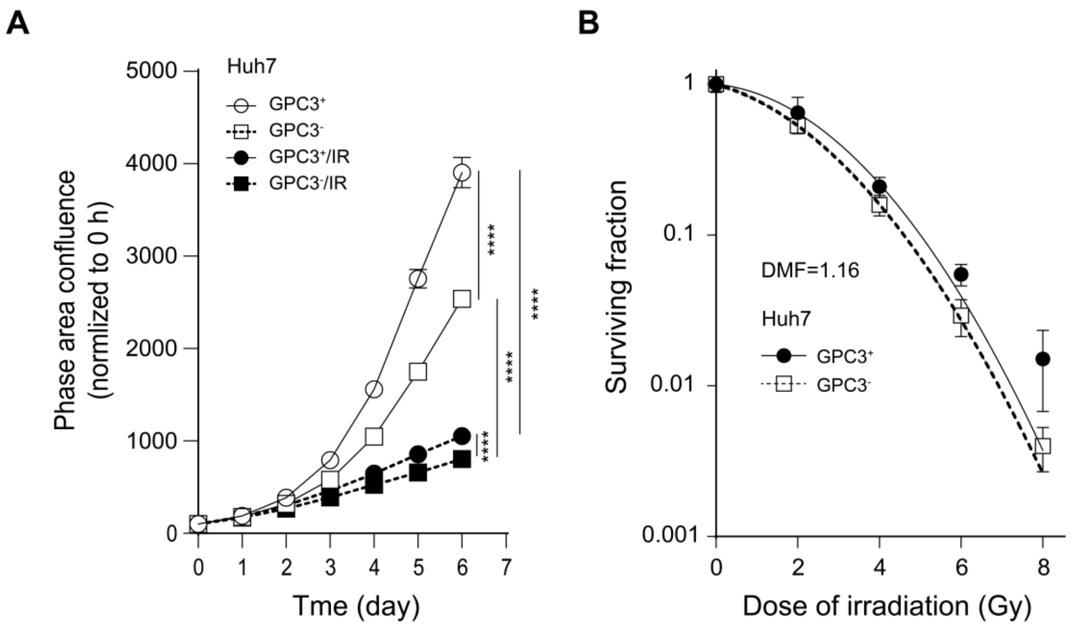

Supplementary Fig. S3. Analysis of proliferation and clonogenic capacity of GPC3^{+} and GPC3^{-} Huh7 cell lines. (*A*) Cell proliferation was monitored using the IncuCyte® live-cell imaging system under control conditions and after 6 Gy IR. (*B*) Clonogenic survival was assessed 10–14 days post-treatment, and survival curves were generated. GPC3-deficient cells showed a modest decrease in colony-forming ability, with a dose modification factor of 1.16 observed in Huh7 cells. Data represent mean survival fractions ± SD from three independent experiments.

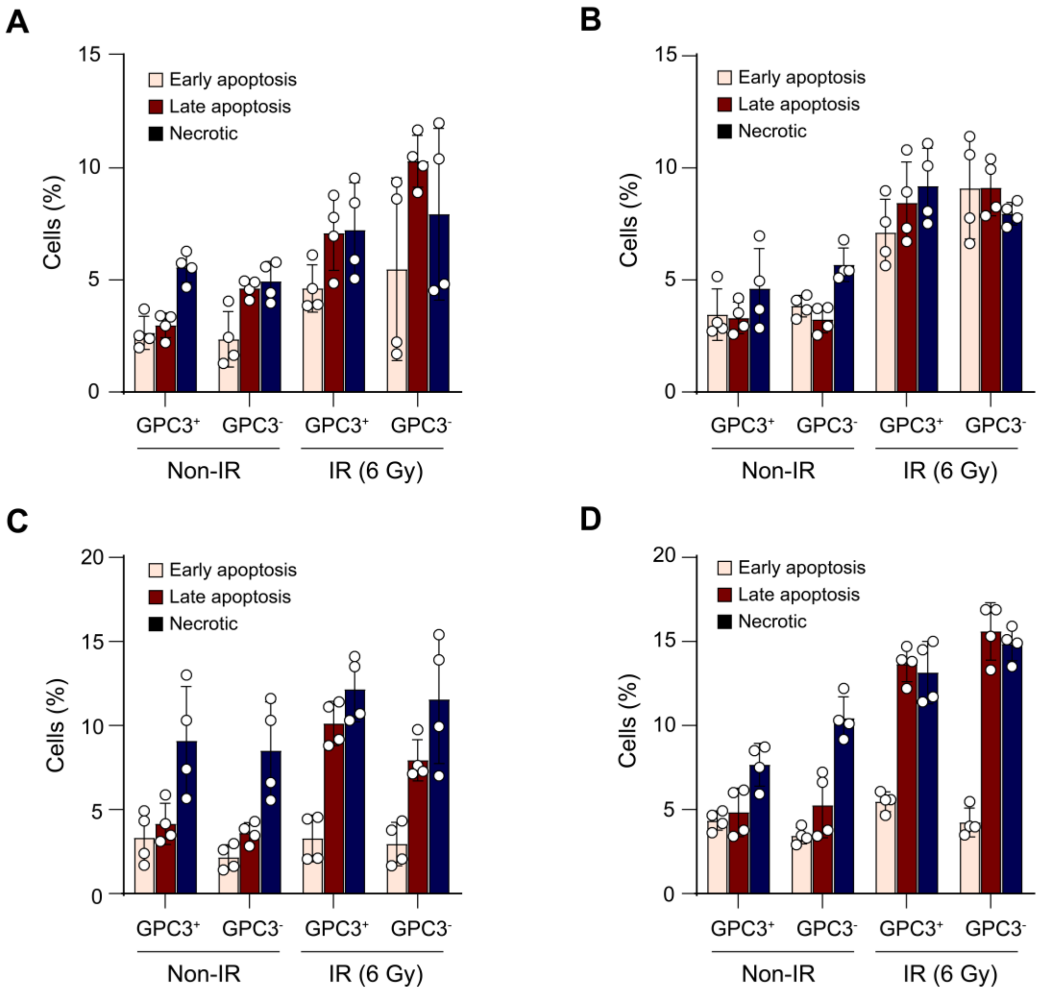

Supplementary Fig. S4. Apoptotic and necrotic profiling of GPC3⁺ and GPC3⁻ liver cancer cells following IR. Flow cytometric analysis was performed to assess apoptosis and necrosis in GPC3⁺ and GPC3⁻ HepG2 cells at 24 h (*A*) and 48 h (*B*) after exposure to 6 Gy of ionizing radiation. A similar analysis was conducted in GPC3⁺ and GPC3⁻ Hep3B cells at 24 h (*C*) and 48 h (*D*) post-irradiation.

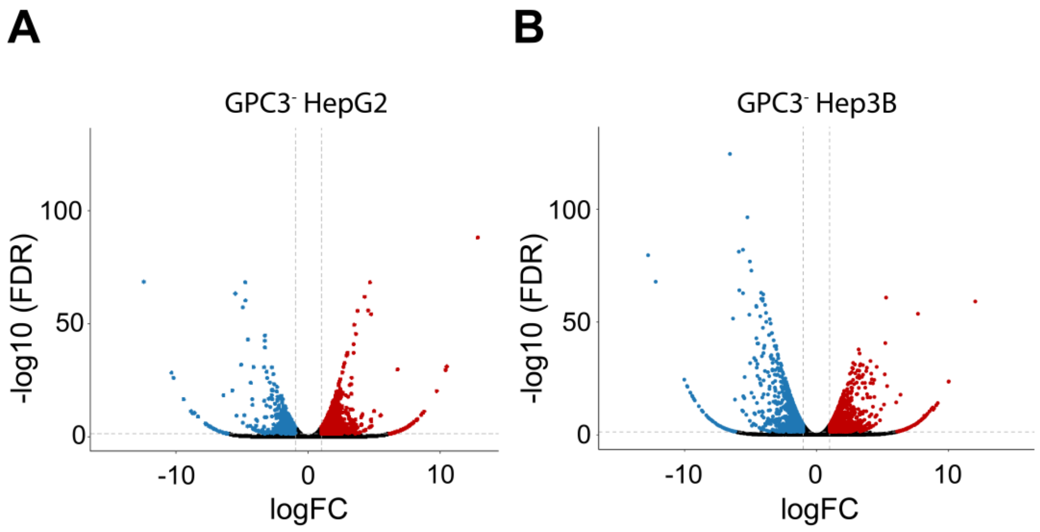

Supplementary Fig. S5. Differential gene expression in GPC3$^-$ liver cancer cell lines. Volcano plots illustrating the differential gene expression profiles in GPC3$^-$ HepG2 (*A*) and Hep3B (*B*) cells compared to their respective parental lines. Each plot displays log2 fold change (x-axis) versus -log10 FDR-adjusted *p*-value (y-axis) for individual genes. Genes with significant downregulation are highlighted in blue, while those with significant upregulation are highlighted in red.

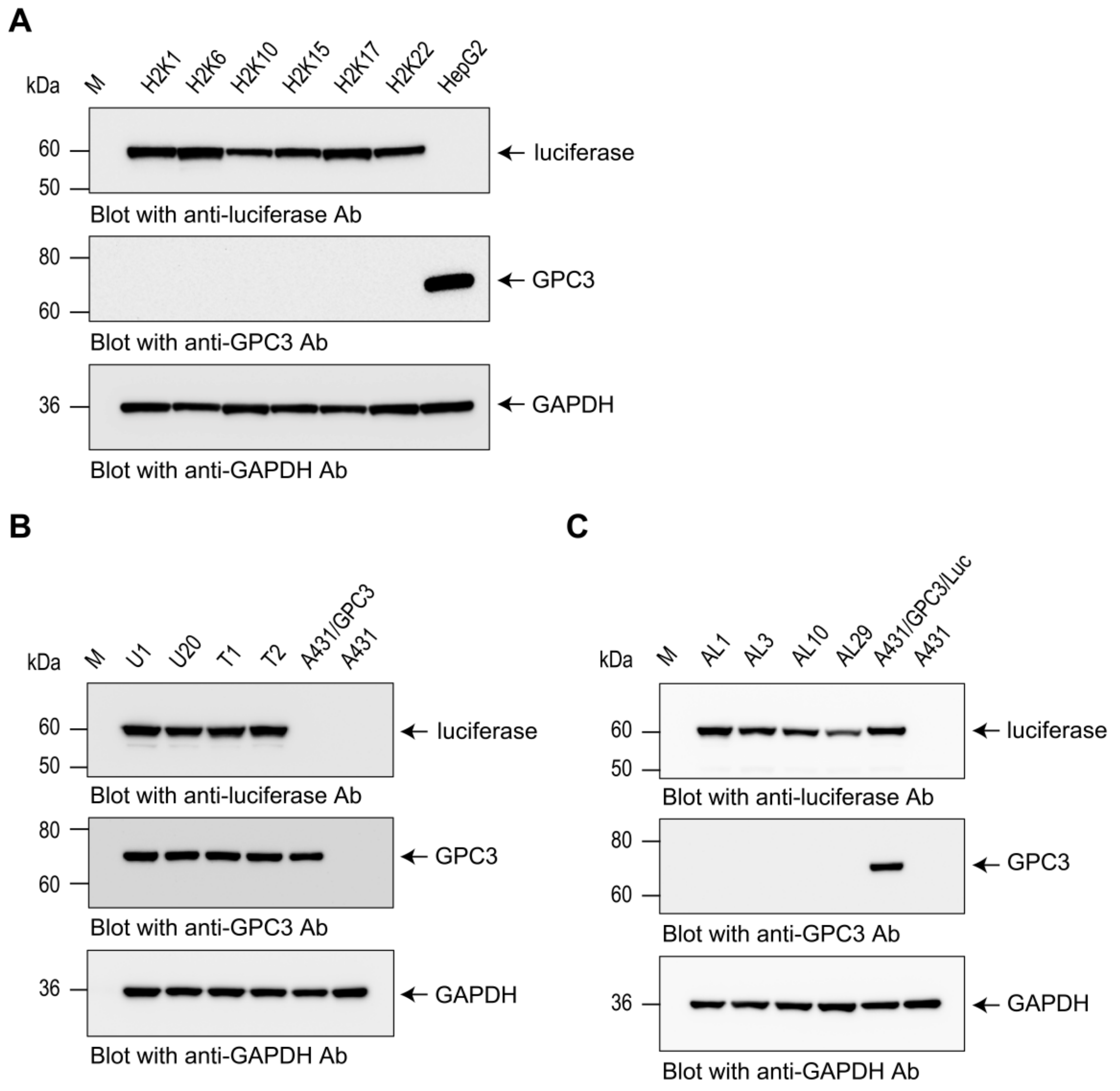

Supplementary Fig. S6. Overexpression of luciferase in GPC3⁻ HepG2, A431/GPC3, and A431 cells using a lentiviral system. Single-cell clones H2K17 (*A*), T2 (*B*), and AL1 (*C*) were selected for GPC3⁻ HepG2, A431/GPC3, and A431 cells, respectively. These clones were used for *in vivo* experiments. GAPDH was used as a loading control.

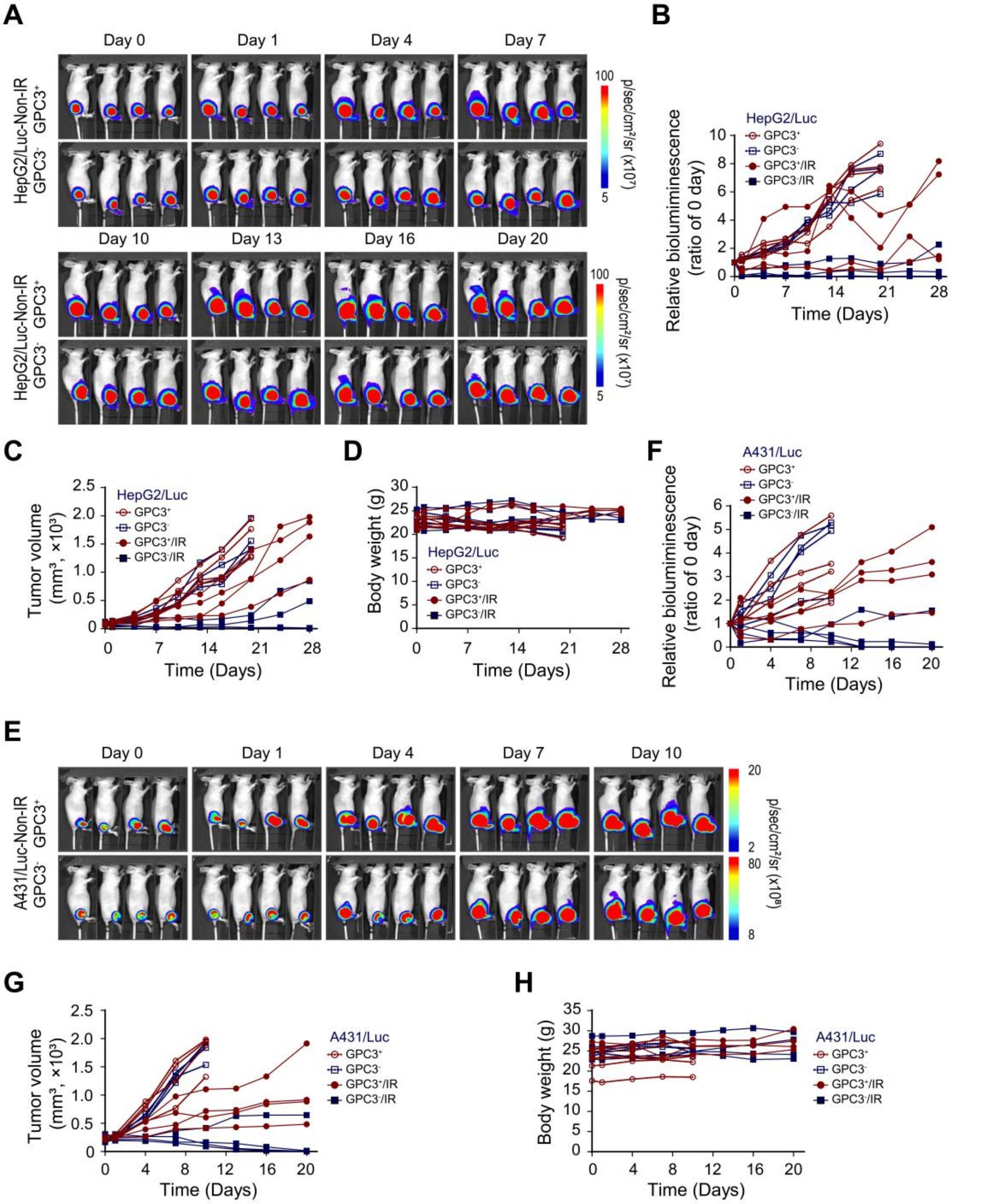

Supplementary Fig. S7. Tumor progression and body weight monitoring in mice bearing GPC3+ and GPC3- tumors with and without irradiation (IR). (*A*, *E*) Longitudinal bioluminescence imaging of mice injected with GPC3+ HepG2/Luc, GPC3- HepG2/Luc, GPC3+ A431/Luc, or GPC3- A431/Luc cells, under non-IR conditions. (*B*, *F*) Quantification of bioluminescence signal over time in the same cohorts, under no-IR and IR conditions. (*C*, *G*) Tumor growth curves estimated by caliper measurements. (*D*, *H*) Body weight changes over time in mice injected with GPC3+ HepG2/Luc, GPC3- HepG2/Luc, GPC3+ A431/Luc, or GPC3- A431/Luc cells.

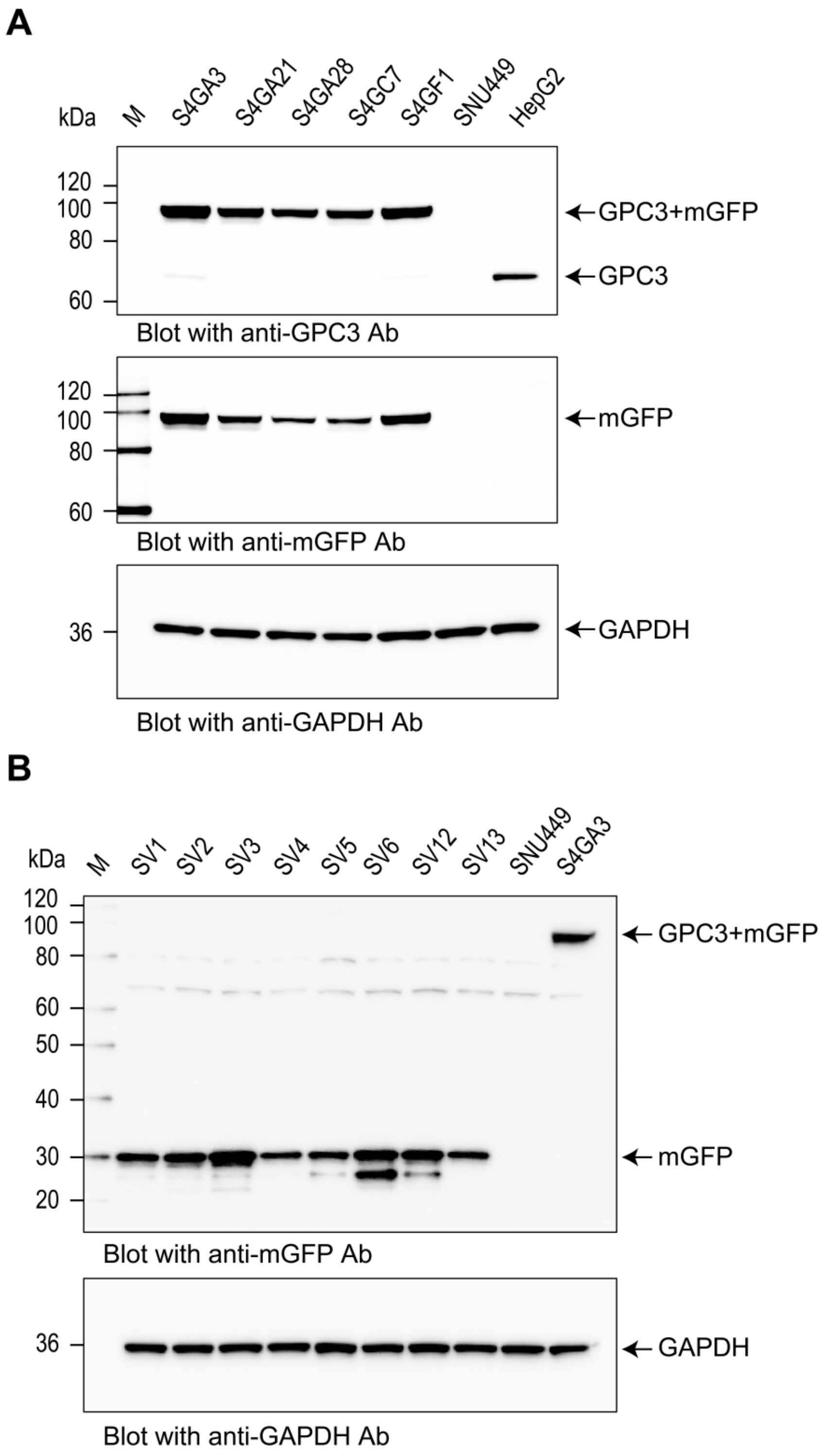

Supplementary Fig. S8. Overexpression of GPC3 in SNU449 cells using a lentiviral system. Single-cell clones S4GA3 (*A*) and SV3 (*B*) were selected for GPC3 overexpression and lentiviral vector control (vehicle), respectively. These clones were used for subsequent analyses. GAPDH was used as a loading control.

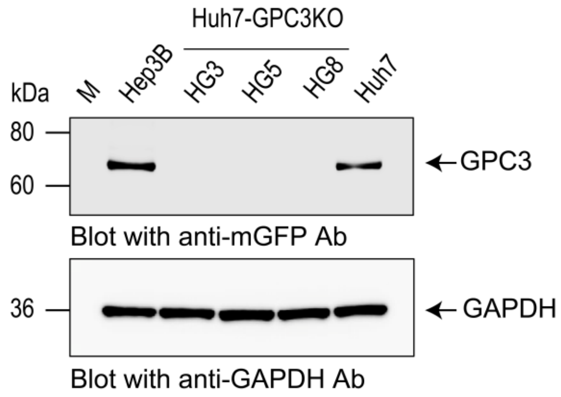

Supplementary Fig. S9. CRISPR/Cas9-mediated knockout of GPC3 in Huh7 cells. The HG3 clone was selected for further experiments.

Supplementary Table S1. Clinicopathological characteristics, GPC3 expression, and radiation therapy details of study cohort

No.	Age	Sex	Growth type	T stage	N stage	Cirrhosis	Steatosis	HBV	HCV	GPC3 expression	RTx (total dose/fraction)
1	72	M	Expanding nodular	T3	N0	-	+	-	-	-	3DCRT (30 Gy/10)
2	62	M	Nodular with perinodular expansion	T2	N0	+	-	+	+	-	3DCRT (39 Gy/11)
3	76	M	Expanding nodular	T3	N0	-	+	-	-	-	SBRT (50Gy/5)
4	53	M	Nodular with perinodular expansion	T1	N0	+	+	+	-	+	3DCRT (39 Gy/13)
5	56	M	Expanding nodular	T2	N0	-	-	+	-	-	3DCRT (39 Gy/13)
6	58	M	Expanding nodular	T3	N0	+	+	+	-	+	IMRT (50 Gy/25)
7	57	M	Expanding nodular	T3	N0	+	-	+	-	+	3DCRT (45 Gy/18)
8	61	M	Nodular with perinodular expansion	T2	N0	+	+	-	-	-	IMRT (50 Gy/25)

HBV, hepatitis B virus; HCV, hepatitis C virus; 3DCRT, three-dimensional conformal radiation therapy; SBRT, stereotactic body radiation therapy; IMRT, intensity-modulated radiation therapy
